# Occult gallbladder carcinoma presenting as a primary ovarian tumor in two women: two case reports and a review of the literature

**DOI:** 10.1186/1752-1947-4-202

**Published:** 2010-06-30

**Authors:** Yashwant Kumar, Alka Chahal, Monika Garg, Anjali Bhutani

**Affiliations:** 1 The Pine, Near Ashiana Regency, Chhota Shimla, Shimla -171002, India; 2 Department of Pathology and Laboratory Medicine, Grecian Superspeciality, Cardiac and Cancer Hospital, Sector 69, SAS Nagar, Mohali, India; 3 Department of Pathology, Maharshi Markandeshwar Institute of Medical Sciences and Research, Mullana, Ambala Haryana, India

## Abstract

**Introduction:**

The ovary is a common site of metastasis from various organs. However, little is known about gallbladder carcinoma metastasizing to the ovaries and presenting as a primary ovarian tumor.

**Case presentation:**

We report two cases of a metastatic gallbladder carcinoma which mimicked a primary ovarian tumor in a 35-year-old and a 62-year-old North Indian woman. Clinically, both our patients presented with abdominal masses without obvious signs and symptoms related to gallbladder carcinoma. Radiology suggested the possibility of a primary ovarian tumor with chronic cholecystitis and cholelithiasis. The gross features also mimicked a primary malignant ovarian tumor in the first case and a benign mucinous neoplasm in the second case. Exact diagnoses could only be made after thorough sampling from both the ovaries and gallbladder.

**Conclusions:**

Gallbladder carcinoma with metastasis to the ovaries can mimic both malignant and benign primary ovarian tumors. Extensive cystic change in the ovary due to metastasis from gallbladder carcinoma has rarely been reported. A high index of suspicion and thorough sampling are essential to avoid misdiagnosis in such cases.

## Introduction

Ovary is a relatively frequent site of metastasis from various organs especially pancreas and gastrointestinal tract. Rarely, the metastasis may precede detection of the primary site and may present as an ovarian tumor [1]. Metastasis from gallbladder to ovaries, though known, is rare with only few reports available in the English literature [2-9]. Some of these were initially misdiagnosed as a primary ovarian tumor. Lack of awareness or limited information may be the reasons for incorrect diagnosis in these cases. Therefore the unique features of occult gallbladder cancer going to ovary need to be explored and reported. Here we describe two such cases that were missed on initial examination. A review of literature has been carried out to search for the most important features which will aid in arriving at a correct diagnosis.

## Case presentation

### Case 1

#### Clinical findings

A 35-year-old North Indian woman presented with abdominal pain and discomfort with loss of appetite and indigestion for one month. Systemic examination revealed abdominal distension and slight tenderness in her right hypochondrium along with palpable bilateral adnexal masses. There was no icterus, but mild elevation of serum bilirubin with normal liver enzyme levels. An ultrasound examination of her abdomen showed a diffusely thickened gallbladder with multiple calculi and bilateral large, solid-cystic adnexal masses suggestive of a primary ovarian malignancy with chronic cholecystitis and cholelithiasis. Her serum tumor marker CA-125 was raised (267.4 U/mL, reference range 0-36 U/mL). Our patient underwent total abdominal hysterectomy and bilateral salpingo-oophorectomy with cholecystectomy. On exploration during surgery the gallbladder was found to be inflamed and adherent to part of omentum, therefore extended omentectomy was performed with removal of pelvic and retro-pancreatic lymph nodes.

#### Histopathology findings

**Figure 1:**
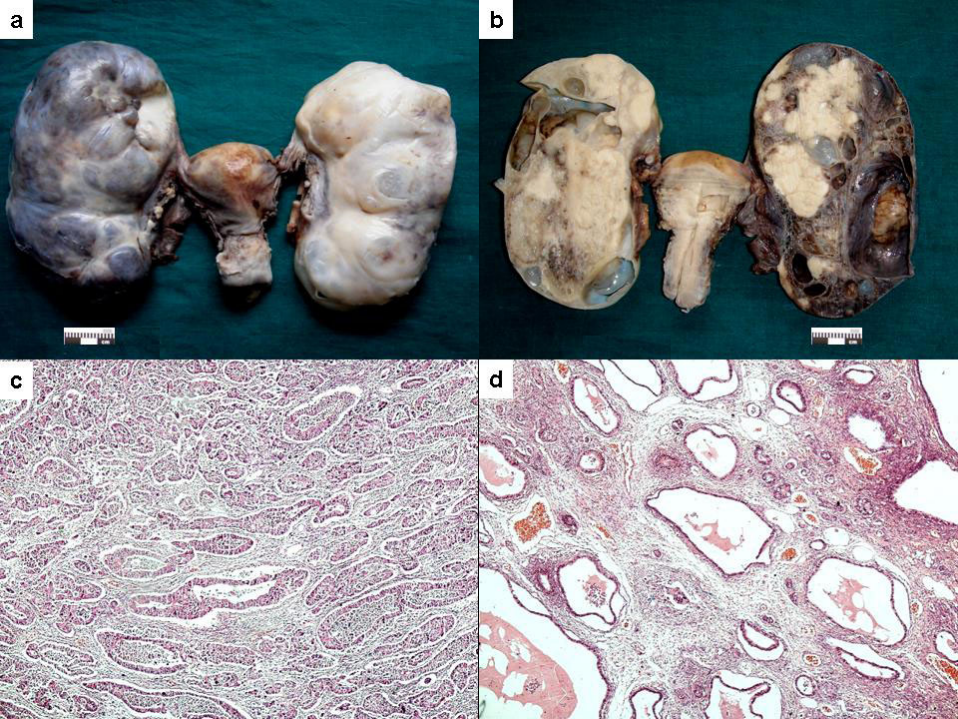
**(A) Capsular surface of bilateral ovarian masses**. Note the smooth looking but nodular outer surface. Also note size of both the masses compared to uterus. **(B) **Cut surface of a solid cystic growth with solid grey-white areas present in the form of nodular deposits. **(C) **On microscopy tumor glands were forming glands of variable size and shape. **(D) **Tumor tissue represented by large cystic spaces lined by flattened epithelium. Smaller glands are also present in between.

Both her right and left ovaries were enlarged and measured 17 × 8 × 5 cm and 16 × 7 × 5 cm, respectively. External surface of both was nodular (Figure 1a) and slicing revealed the parenchyma almost completely replaced by a tumor with involvement of hilum as well. The cut surface was multinodular and had a variegated appearance with both solid and cystic areas. Solid areas were well demarcated, soft to firm and pale-yellow in color. The cystic spaces were filled with mucinous material (Figure 1b). Bilateral fallopian tubes, uterus and cervix were normal.

Both the masses showed a similar morphology on microscopy. Solid areas were composed of irregular glands and nests infiltrating the loose stroma (Figure 1c). The tumor was reaching up to capsule and encroaching upon the surface. The glands were lined by large pleomorphic cells exhibiting high grade nuclear atypia. Cystic areas showed dilated spaces lined by malignant cells (Figure 1d). Bizarre tumor giant cells, occasional signet ring cells and atypical mitotic figures were noted. Large areas of infarction and necrosis were also seen. Normal ovarian stroma was identified in one of the sections only.

**Figure 2:**
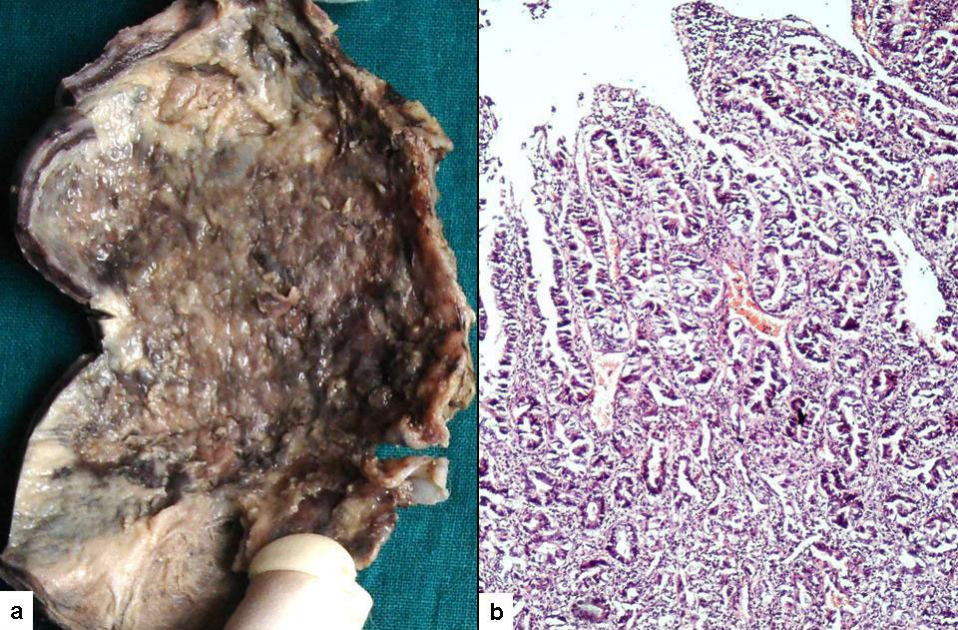
**(A) Diffusely hemorrhagic and ulcerated gallbladder mucosa**. No growth is apparent. **(B) **An invasive adenocarcinoma with dysplastic overlying epithelium.

The gallbladder had a gangrenous appearance with diffusely hemorrhagic and thickened wall covered with slough on both the serosal as well as mucosal aspect (Figure 2a). The lumen contained multiple mixed stones. Besides extensive necrosis and hemorrhage, sections from viable areas showed an invasive adenocarcinoma with transmural involvement of the wall and overlying dysplastic epithelium (Figure 2b). Perineural invasion was also noted. The omentum and retro-pancreatic lymph nodes showed tumor metastasis in the form of pools of mucin infiltrating and dissecting the native tissue. The tumor cells were found to be floating within the mucin and many of them had a signet ring appearance.

### Case 2

#### Clinical findings

**Figure 3:**
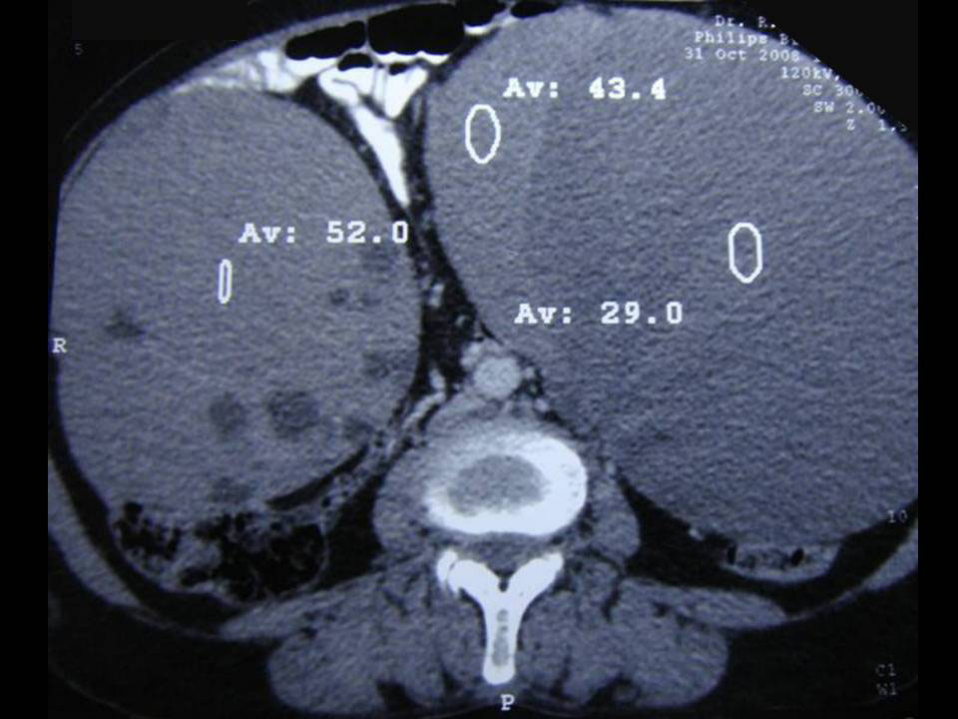
**CT scan of abdomen showing two large cystic masses arising from pelvis**.

A 62-year-old woman from a Northern part of India presented with complaints of pain and swelling in the abdomen and generalized weakness for a duration of four months. Routine biochemistry including liver function tests and hematological parameters were normal. A computed tomography (CT) scan of her abdomen showed two large masses arising from pelvis on either side of the uterus. The masses were reaching up to epigastrium and displacing gut loops anteriorly and towards right side. Both of them were largely cystic with well defined walls (Figure 3). Her gallbladder contained multiple stones and wall in the fundic region was thickened resembling calcification. There was no ascitis or pleural effusion and CA-125 was raised (148.2 U/mL). Radiological impression was cholelithiasis and bilateral ovarian tumor of benign nature. However, considering the age of our patient, size of the masses and raised CA-125 it was thought to be an ovarian malignancy and exploratory laparotomy was done for total abdominal hysterectomy with bilateral salpingo-oophorectomy and cholecystectomy. Intra-operative findings revealed bilateral cystic ovarian masses and a hard and solid gallbladder mass firmly adherent to surrounding tissue. Omental nodules were also noted and removed.

#### Histopathology findings

**Figure 4:**
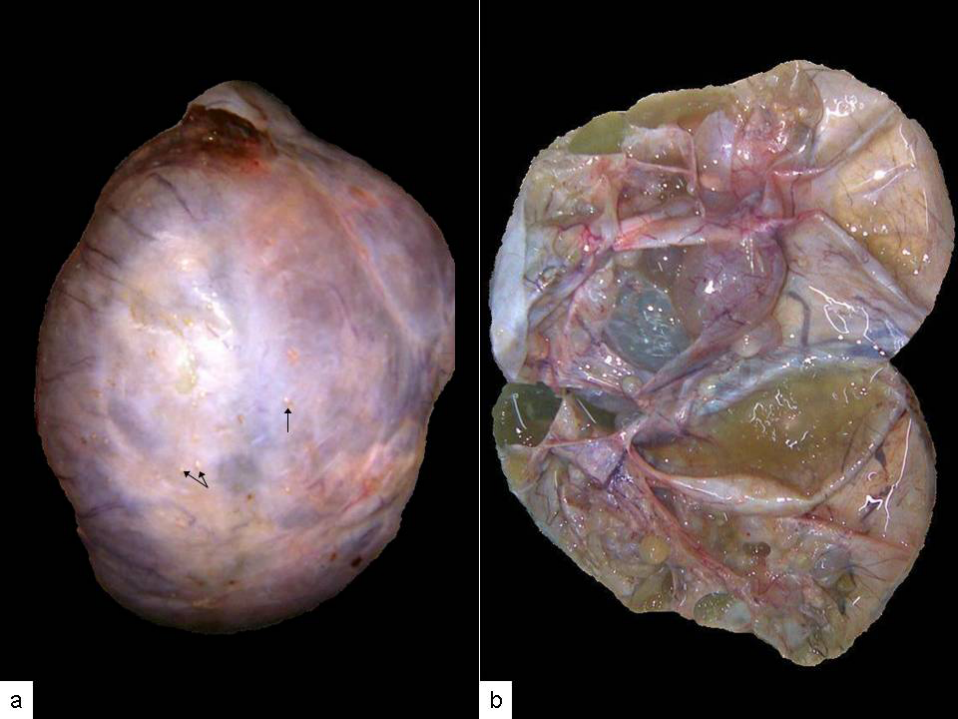
**(A) Well encapsulated left ovarian mass**. Note tiny pinhead size excrescences on the surface (arrow). **(B) **The cut surface resembling a multiloculated benign cystic tumor.

Bilateral ovarian masses were well encapsulated with right mass measuring 20 × 18 × 11 cm and left 18 × 13 × 10 cm. Capsular surface of both revealed evenly distributed multiple tiny pinhead size excrescences (Figure 4a). Cut surface revealed multiloculated cystic tumor filled with thick and solidified gelatinous material as well as dull colored fluid (Figure 4b). The septae were papery thin, at places forming small cysts giving a spongy appearance. No solid areas were found in either of the masses even on serial slicing except two very small 0.5 cm diameter, subcapsular grey-white nodules. Her uterus showed an incidental 1.5 cm intra-mural leiomyoma in the fundic region. Her cervix, bilateral fallopian tubes and ovarian pedicles were normal.

**Figure 5:**
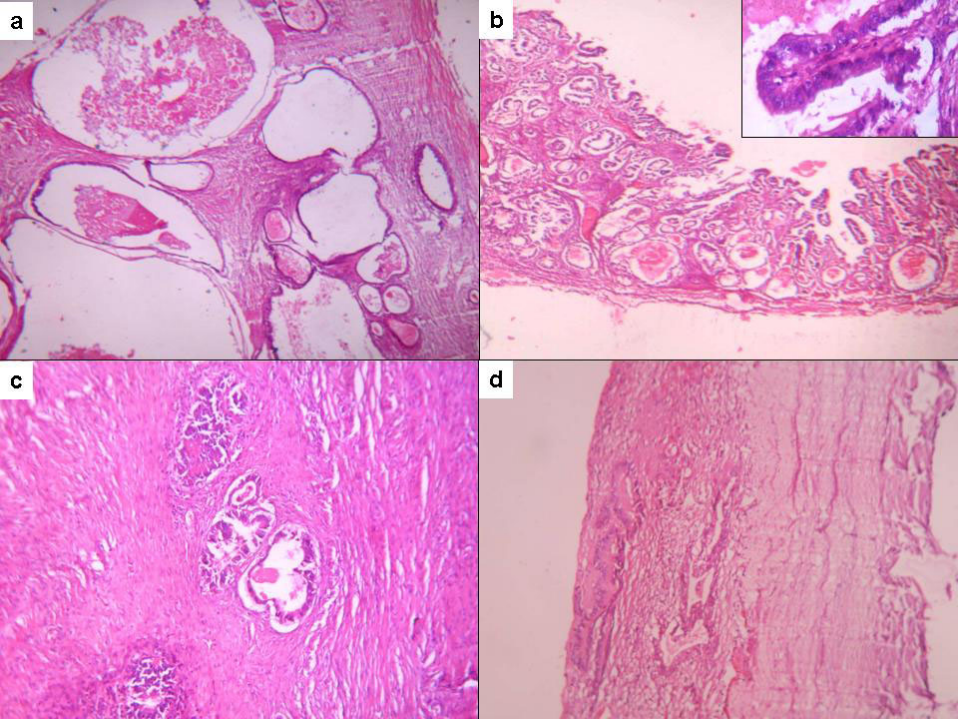
**(A) Large cystic spaces lined by flattened epithelium and filled with acellular material**. **(B) **Malignant tumor glands with back to back arrangement. Note marked atypia of cells within papillae (inset). **(C) **Irregular shaped glands within the desmoplastic stroma. **(D) **Surface implants.

On microscopy cystic spaces were lined by flattened epithelium and filled with acellular material (Figure 5a). On low power examination lining epithelium was flattened to columnar and appeared bland without any stratification or multilayering. Therefore the possibility of benign mucinous cystadenoma was initially proposed. The additional sections however revealed marked atypia of the lining epithelium. Two out of 23 sections taken from small subcapsular nodules showed atypically proliferating mucinous epithelium (Figure 5b). Few papillae were also seen lined by epithelial cells with marked atypia. Intervening stroma was scanty but few foci of infiltration by irregular shaped glands were identified (Figure 5c). Tiny excrescences present on the capsular surface showed tumor gland deposits (Figure 5d) supporting the possibility of a metastatic tumor. Uninvolved ovarian parenchyma was fibrous and contained hemosiderin laden and foamy macrophages.

**Figure 6:**
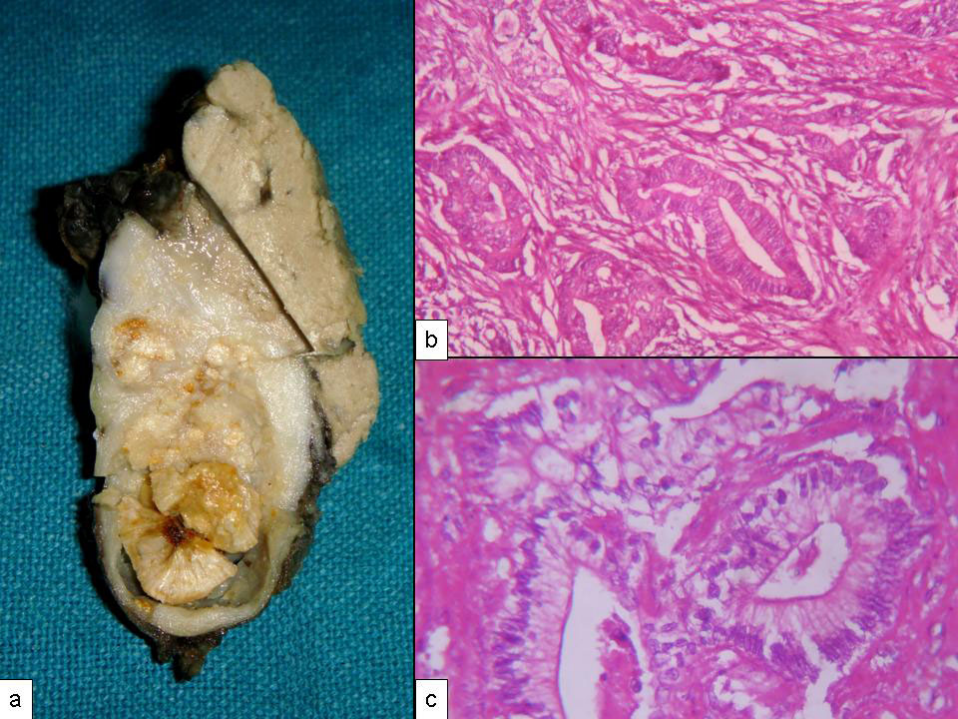
**(A) Thickened gallbladder wall with a fragmented stone**. **(B) **Well formed tumor glands within a desmoplastic stroma. The glands are lined by columnar cells with basally placed nuclei.

The serosal surface of gallbladder was smooth and shiny. The lumen was impacted with a 1.3 cm diameter cholesterol stone. In the body region mucosa was ulcerated with variably thickened wall (Figure 6a). Microscopy showed a moderately differentiated adenocarcinoma (Figure 6b). Omental nodules showed metastatic tumor deposits with a similar morphology as in case 1.

## Discussion

The incidence of ovarian metastasis from different organs is nearly five to 15% [7]. Although a figure of 6% cases of gallbladder carcinoma with metastasis to ovary has been quoted by Albores-Saavedra [10], a description of only 19 such cases could be found in the literature (Table 1) [2-9]. Of these, eight cases presented with ovarian masses [2,3,5,6,8,9] and clinico-radiological findings in five mimicked a primary ovarian tumor [2,3,8]. With a pre-operative radiological investigation, diagnosis could not be established in four cases [4-7] and few were misdiagnosed as primary ovarian tumor even on histology [6,7].

**Table 1:** A summary of reported cases of gallbladder carcinoma with ovarian metastasis.

Author	No. of cases	Age (yrs)	Clinical presentation	Detection of primary/secondary	Laterality	Size (cm)	Histopathology of ovary	
							Gross	Micro
Khunamornpong *et al*.[2]	8	47-83	Pelvic mass, abdominal distension, vaginal bleeding, hematochezia n = 1 each abdominal pain, unknown n = 2 each	Primary first n = 3 Simultaneous n = 5	Bilateral	0.5-16.5	Smooth external surface in majority, cut surface predominantly solid-cystic or solid in some, cyst content mucoid in majority	All except 1 were recognized as metastatic tumors; initially diagnosis was not appreciated in 1 case. All had foci indistinguishable from primary surface epithelial neoplasms
Young and Scully[3]	5	33-72	Abdominal pain n = 4 Pelvic mass n = 1	Primary first n = 1 Simultaneous n = 3 Ovarian first n = 1	Bilateral	2.5-13	Lobulated external surface. Cut surface in all except 1 was nodular and solid	Half of them were difficult to diagnose and simulated primary ovarian neoplasm
Ayhan *et al*.[4]	1	33	Abdominal pain	Simultaneous	Unilateral	3	-	-
Miyagui *et al*.[5]	1	43	Confusion	Simultaneous	Bilateral	17 and 19	Cut surface compact intermingled with cystic areas containing yellow gelatinous fluid	Ovarian architecture entirely replaced neoplastic cells disposed in alveolar and trabecular patterns. Mucin & signet ring cells
Jain *et al*.[6]	1	45	Pelvic mass	Simultaneous	Bilateral	-	-	Malignant cystic deposits
Jarvi *et al*.[7]	1	82	Abdominal pain	Simultaneous	Bilateral	-	Solid cystic masses with focally roughened surfaces	Bilateral benign serous cystadenoma with deposits of metastatic adenocarcinoma
Taranto *et al*.[8]	1	52	Pelvic mass	Primary first	Bilateral	15	-	Difficult to distinguish from a primary mucinous adenocarcinoma of the ovary even on histology
Majumdar *et al*.[9]	1	38	Abdominal pain and distension	Simultaneous	Bilateral	13 and 8	-	Papillary pattern, cystic spaces, extracellular mucin, surface implants
Kumar *et al*. (present study)	2	35 62	Abdominal pain Abdominal pain and distension	Simultaneous	Bilateral	17 and 18 20 and 18	Case 1: Solid cystic masses and gangrenous gallbladder Case 2: Entirely cystic, multiloculated ovarian masses filled with thick and thin mucin	Nodular growth with infiltrative pattern. Presence of surface deposits, cellular atypia, and infiltrative pattern

Similar to the present report, a majority of such patients had non-specific abdominal or pelvic symptoms (pain, distension, or mass). Jaundice or other symptoms related to gallbladder carcinoma were observed in only few cases [2,6,9]. Radiological features of malignancy were masked by chronic cholecystitis or cholelithiasis. Serological markers such as alkaline phosphatase, CA19-9, CEA, and CA-125 were found to be variable at the time of metastases [2-4,6-9]. In both our patients CA-125 levels were raised, however CA19-9 was not assessed. A variable clinical presentation, radiology and serum markers make the appropriate histological diagnosis mandatory [3,11-13].

The morphological features, on histology of metastasis, may mimic not only malignant but also a benign ovarian tumor as observed in our patients. In the first case, the gallbladder was gangrenous and no obvious growth was apparent on gross examination. Microscopically, only a few tumor glands were noticed in one of the sections taken from the gallbladder. The origin of these glands could not be traced from these initial sections. The gallbladder therefore was re-grossed. Repeat sections taken revealed a tumor diffusely involving the gallbladder wall with overlying dysplastic epithelium. This along with a bilateral tumor, multinodularity, infiltrative pattern and presence of uninvolved tissue supported the possibility of a metastatic carcinoma rather than a primary malignancy in the ovaries.

The second case showed a full-fledged gallbladder malignancy. The ovarian masses, however, were completely cystic with no solid areas. The initial sections suggested possibility of a benign mucinous tumor. However, presence of focal atypia in the lining epithelium and a high index of suspicion, in view of presence of a gallbladder malignancy led to re-examination of the specimen. Tiny pinhead size elevations over the capsule (Figure 3b) and subcapsular nodules identified on second look revealed malignant glands, which supported the possibility of a metastatic tumor.

In the literature a variety of features have been emphasized (Table 2) that may help to differentiate metastasis from a primary ovarian tumor [2,11,14]. Amongst these, the bilaterality, surface implants, multinodularity, infiltrative pattern, foci of uninvolved ovarian tissue, growth in the ovarian hilum, mucin without epithelial cells on the tumor surface and presence of signet ring cells are the most important clues for a metastatic adenocarcinoma. However, many of these features may be absent, especially if the metastasis presents as benign cystic mass. Although the immunohistochemistry can distinguish metastasis from other organs with respect of colorectal carcinoma (CK7^-^/CK20^+^) in contrast to ovarian primaries (CK7^+^/CK20^-^/CK20^+^), its role in metastasis from gallbladder is limited because of similar profile to that of primary ovarian mucinous tumors [2,15]. A thorough gross examination and adequate sectioning therefore are important in such cases.

**Table 2:** Pathological features differentiating a secondary from primary ovarian tumor [2,11,14,15]

Pathological features	Secondary	Primary
**Gross**		
Bilaterality	✓	
Surface implants	✓	
Multinodular growth	✓	
Size > 10 cm	✓	✓
Smooth tumor surface		✓
Mural nodule		✓

**Micro**		

Surface implants in the form of irregular/dilated/cystic/angulated/tubular glands/cell nests or single tumor cells within a desmoplastic/hyalinized stroma	✓	
Infiltrative pattern (disorderly penetration of the stroma by small glands, tubules, or single cells, including signet-ring cells, usually within a desmoplastic stroma)	✓	
Growth in the ovarian hilum	✓	
Foci of uninvolved ovarian tissue	✓	
Mucin without epithelial cells on the tumor surface or the residual ovarian surface	✓	
A predominantly cystic gross appearance with only few solid necrotic or hemorrhagic areas	✓	✓
Grossly mucinous cyst contents	✓	✓
Areas of a cribriform, villous, or solid growth	✓	✓
Microscopic mucin extravasation into the stroma	✓	✓
Benign or borderline-appearing areas (either with atypia only or with intraepithelial carcinoma)	✓	✓
Focal endometrioid-like appearance	✓	✓
Microscopic cysts, generally > 2 mm	✓	✓
"Expansile" invasive pattern (sharply demarcated, multicystic or labyrinthine spaces lined by malignant-appearing epithelial cells, with minimal or no recognizable intervening stroma, in an area exceeding 10 mm and at least 3 mm in any single dimension)		✓
A complex papillary epithelial growth (branching papillae with epithelial stratification and little or no stromal support)		✓
Intraluminal necrotic material (tumor cell karyorrhectic nuclear fragments, neutrophils, and acellular debris) in gland-cyst lumens		✓

**Immunohistochemistry**		

CK-7	✓	✓
CK-20	✓	✓
Dpc4	✓	✓

Outcome in these cases is generally poor. However, adequate surgery with palliative treatment may prolong survival for few months. Therefore at the time of total abdominal hysterectomy and bilateral salpingo-oophorectomy with cholecystectomy presence of unusual findings such as a gallbladder mass, dense adhesions of the omentum and adjacent organs to the gallbladder, difficult dissection of the gallbladder from its liver bed should raise the suspicion of a carcinoma. A close evaluation of the extent of the disease should be carried out. Biopsy of any lymph node should be taken. Intra-operative ultrasound, intra-portal endoscopic ultrasound and frozen section all may be performed to assess the extent of the disease. In the presence of ascites, fluid should be obtained for cytology; otherwise, a peritoneal wash-out can be considered for cytology [16]. External radiation therapy with or without chemotherapy may provide some palliative benefit to these patients.

## Conclusions

Gallbladder carcinoma should be added to the previously known list of origins of metastatic tumors to the ovary that can closely mimic primary ovarian mucinous tumors. Pathologists should maintain a high index of suspicion and adequate sampling should be done of ovarian masses especially if bilateral. In all bilateral mucinous tumors outer surface should be examined carefully for presence of tiny deposits. Knowledge of the extent to which gallbladder metastasis may mimic a primary ovarian tumor and its differentiating histological features may help in correct diagnosis and further management of the patient.

## Consent

Written informed consent was obtained from both the patients for publication of this case report and any accompanying images. A copy of the written consent is available for review by the Editor-in-Chief of this journal.

## Competing interests

The authors declare that they have no competing interests.

## Authors' contributions

YK designed, carried out acquisition and analysis of data and drafted the manuscript. AC and AB helped in drafting of manuscript and given their valuable suggestions, MG provided the images. All the authors read and approved the final manuscript.
